# Accelerated skeletal maturation is associated with overweight and obesity as early as preschool age: a cross-sectional study

**DOI:** 10.1186/s12887-020-02353-w

**Published:** 2020-09-28

**Authors:** Dandan Ke, Dajiang Lu, Guang Cai, Jing Zhang, Xiaofei Wang, Koya Suzuki

**Affiliations:** 1 Graduate School of Health and Sports Science, Juntendo University, 1- 1 Hiraka-gakuendai, Inzai-city, Chiba 270-1695 Japan; 2 School of Kinesiology, Shanghai University of Sport, No. 650 Qingyuan Ring Road, Yangpu District, Shanghai, 200438 People’s Republic of China; 3 Shanghai Research Institute of Sports Science, No.87 Wuxing Road, Xuhui District, Shanghai, 200030 People’s Republic of China; 4 Shanghai Center for Women and Children’s Health, No.339 Luding Road, Putuo District, Shanghai, 200062 People’s Republic of China

**Keywords:** Pediatric obesity, Relative skeletal age, Advanced bone age, Growth and maturation, Body mass index

## Abstract

**Background:**

Body mass index (BMI) and skeletal age (SA) are important indicators of individual growth and maturation. Although the results have not been unified, most studies indicated that accelerated skeletal maturation is associated with overweight/obesity. However, there have so far been insufficient studies about the association between accelerated skeletal maturation and overweight/obesity in preschoolers, particularly Asian children. A cross-sectional study was conducted on Chinese children to verify the association between accelerated skeletal maturation and overweight/obesity at preschool age.

**Methods:**

The study involved 1330 participants aged 3.1–6.6 years old (730 males and 600 females) in Shanghai, China. The skeletal age was determined according to the method of TW3-C RUS. Accelerated skeletal maturation was defined as relative SA (SA minus chronological age [CA]) ≥1.0 years. BMI was classified as thinness, normal weight, overweight, and obesity according to the International Obesity Task Force (IOTF) BMI cut-offs. The Chi-square was performed to determine the statistically significant difference in the frequency of accelerated skeletal maturation in BMI and age categories. The logistic regression model analyzed the association between accelerated skeletal maturation and overweight/obesity.

**Results:**

The percentage of accelerated skeletal maturation increased with BMI (7.8% of children in thinness group had accelerated skeletal maturation; the percentage increased to 30.8% in obese group. *x*^*2*^ *= 89.442, df = 3, P < 0.01*) and age group (at age 3.5, 3.5% of participants had accelerated skeletal maturation; at age 6.0 years, this increased to 27.8%. *x*^*2*^ *= 43.417, df = 5, P < 0.01*). Logistic regression analysis showed that children with overweight and obesity are more likely to have accelerated skeletal maturation than children with normal weight after adjusting for gender and age (Overweight, odds ratio *[OR] = 3.27, 95%* confidence interval *[CI]: 2.20–4.87;* Obese, *OR = 4.73, 95% CI: 2.99–7.48*).

**Conclusions:**

There is an association between accelerated skeletal maturation and overweight/obesity among preschool children. This study suggests that accelerated skeletal maturation might coexist with overweight/obesity in preschool children, and interventions, such as dietary modifications and increasing levels of physical activity, should be employed to prevent both accelerated skeletal maturation and overweight/obesity as early as preschool age.

## Background

Overweight and obesity are defined as abnormal or excessive body fat accumulation that may impair health [1], and the BMI is widely used to screen for overweight and obesity. It has previously been observed that the prevalence of childhood overweight and obesity is increasing worldwide recently [2], and the American Academy of Pediatrics stated that overweight and obesity are currently the most common medical conditions of childhood [3]. Compared to children with normal-weight, children with overweight have at least twice the risk of becoming overweight adults [4]. In addition to increased future risks, children with obesity experience breathing difficulties, increased risk of fractures, hypertension, early markers of cardiovascular disease, insulin resistance, and psychological effects [1].

Skeletal age (SA), which is distinct from chronological age (CA), an indicator of physiological maturation, is commonly used in children and adolescents (above 3 years old) for excluding underlying disorders involving the growth hormone or sex steroid pathways, such as early or late puberty [5], hypertension [6], skeletal dysplasia, short or tall stature, growth hormone deficiency, and congenital adrenal hyperplasia [7, 8]. The relative SA (SA minus CA) indicates the skeletal maturation status of a child, and when it is greater than 1.0 year old, the child is considered to have accelerated skeletal maturation [6, 9, 10]. Accelerated skeletal maturation was considered to result in a decrease in final height by accelerating the early closure of the epiphysis and terminating the child’s growth prematurely [11, 12]; similarly, it was suggested as a risk indicator for the development of obesity by a 15-year longitudinal study [13].

BMI and SA are important indicators of individual growth (height and weight) and maturation (skeletal maturation) [10]. Height/weight and skeletal maturation during childhood and adolescence are both influenced by endocrine and paracrine factors, such as genes, nutrition status, and hormones [14]. Several studies reported that children and adolescents with overweight and obesity are more likely to have accelerated skeletal maturation [15–17]. Instead, results obtained from a study demonstrated no association between overweight or obesity and significantly accelerated skeletal maturation in adolescents [18]. There is one evidence that leptin, the main product of adipose tissue, did not correlate with SA [19]. Similarly, Russell et al. suggested that body adiposity might not be the primary reason for accelerated skeletal maturation [20]. Considering the different outcomes obtained in previous studies, it is still necessary to perform relevant research to verify one of the supporting evidence.

On the contrary, the previous research was mainly conducted among Western children and adolescents. There is minimal evidence about Asian children and adolescence, and the applicability of the conclusion to Chinese children is unknown because of the ethnic differences. To our knowledge, there are fewer studies that showed the evidence on the association between skeletal maturation and BMI in children younger than 6 years. If there is an association between overweight/obesity and accelerated skeletal maturation in preschool children, as the prevalence of overweight and obesity gradually increased in preschool children [2], the occurrence of accelerated skeletal maturation should be a concern from the preschool stage. Early recognition of the association between accelerated skeletal maturation and overweight/obesity could prompt timely obesity management to stop a vicious circle that overweight/obesity and accelerated skeletal maturation reinforce each other. This provides an opportunity to positively affect skeletal maturation before the onset of puberty disorders that can be commonly classified into precocious or delayed puberty [21] (idiopathic central precocious puberty occurs in 74% of females and 60% of males [22]). Therefore, this cross-sectional study aimed to verify the association between accelerated skeletal maturation and overweight/ obesity in preschool children.

## Methods

### Participants

**Fig. 1:**
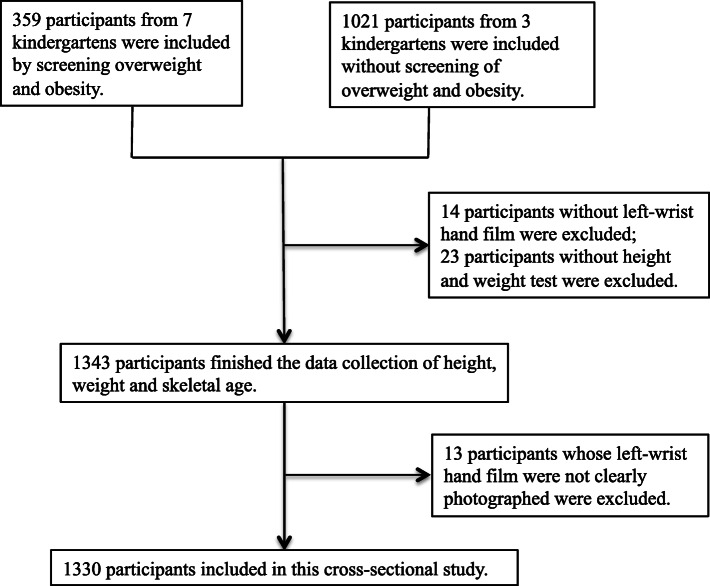
Flowchart of sample selection information

This cross-sectional study was conducted at school entry (autumn 2019) in Shanghai city. Participants were non-randomly selected from 10 public kindergartens rated as demonstration and first-level kindergarten by the Shanghai Kindergarten Education Center based on facilities and environment, child development, education, and health care [23, 24]. The recruitment of the subjects was classified into two parts. The first part was to select 359 participants with overweight/obesity from 7 kindergartens. The screening was performed with the assistance of the kindergarten’s healthcare teacher, referring to the standard that “For children under 5 years of age, weight-for-height greater than 2 standard deviations, and for children over 5 years old, BMI-for-age is greater than 1 standard deviation above the World Health Organization (WHO) Growth Reference median” [1]. The second part was that 1021 children in the other three kindergartens who are willing to participate in this study were included, without considering the body type. Written study information and informed consent forms were sent to the participants’ parents by the study team. In addition, teachers in each kindergarten received the study introduction content. The children were formally included in the study when the informed consent forms were received. After conducting the test of body weight and left hand-and-wrist film, the missing data were excluded; finally, we studied 1330 children aged 3.1 to 6.6 years (730 males, 600 females). The screening process is shown in Fig. 1. The participants included in this study were compared with the Height and Weight Standardized Growth Charts for Chinese Children (updated every 5 years, divided into urban and rural version) established by the China Capital Institute of Pediatrics in 2015, which are widely used in children under 7 years old in China [25].

The weight (kg) and height (cm) of the children were measured to 0.1 kg and 0.1 cm, respectively, without shoes and coats on. In the measurement of body height, the child stood upright on a mechanical stadiometer (Ningbo Finer Medical Instruments Co., Limited, Zhejiang, China) with the heels close together, and the toes approximately 60° apart; the heels, buttocks, and scapulae were in contact with the backboard of the stadiometer, and the head was positioned in a horizontal plane. In the measurement of body weight, the child naturally stood in the center of an OMRON body fat and weight measurement device (V-BODY HBF-371, Omron, Japan) until the data stabilized. Notably, BMI was calculated using the equation below:

$$ \mathrm{BMI}\ \left(\mathrm{kg}/{\mathrm{m}}^2\right)=\mathrm{Weight}\ \left(\mathrm{kg}\right)/\mathrm{Height}\ \left({\mathrm{m}}^2\right) $$

The phenotype was classified into thinness, normal, overweight (excluding children with obesity), and obese groups according to the BMI cut-offs of the IOTF [26] (Table 1).

**Table 1:** Revised IOTF BMI cut-offs (kg/m^2^) using the pooled LMS curves [26]

Age (years)	Males			Females		
	BMI 18.5^a^	BMI 25^a^	BMI 30^a^	BMI 18.5^a^	BMI 25^a^	BMI 30^a^
	Thinness	Overweight	Obese	Thinness	Overweight	Obese
3.0–3.49	14.83	17.85	19.50	14.60	17.64	19.38
3.5–3.99	14.66	17.66	19.33	14.44	17.48	19.25
4.0–4.49	14.51	17.52	19.23	14.30	17.36	19.16
4.5–4.99	14.38	17.43	19.20	14.16	17.27	19.14
5.0–5.49	14.26	17.39	19.27	14.04	17.23	19.20
5.5–5.99	14.15	17.42	19.46	13.93	17.25	19.36
6.0–6.49	14.06	17.52	19.76	13.85	17.33	19.62
6.5–6.99	14.00	17.67	20.15	13.81	17.48	19.96

^a^Indicates BMI centile corresponding to BMI at age 18 using pooled LMS-based cut-offs

*IOTF* International Obesity Task Force, *BMI* Body Mass Index, *LMS* smooth (Lambda), mean (Mu), coefficient of variation (Sigma)

### Skeletal maturation

Radiographs of the left hand and wrist were taken with a digital portable X-ray apparatus (MOVIX4.0 + D Ream, Stephanie, France). Following this, the investigator assisted the children in placing their left palms, face downwards in contact with the film, with the axis of the middle finger directly in line with that of the forearm, and the fingers were naturally separated to remain contactless. Notably, a lead protective box was attached to the device to protect the child.

The skeletal maturation assessment was performed by comparing the obtained X-ray Film with the standard of Tanner–Whitehouse 3-Chinese Radius–Ulna–Short bones (TW3-C RUS) [27]. In total, 13 bones (the proximal, middle and distal phalanges, the first, third and fifth metacarpals, the carpals, and, lastly, the distal radius and ulna) were compared to the standards published in the Atlas of Skeletal Maturation; maturity scores were estimated and converted to SA. The reliability of SA evaluation was analyzed following the method used in a study by Chaumoitre et al. [28]. In this study, the radiographs were analyzed by the same observer following the standard of TW3-C RUS. To evaluate inter- and intra-observer variations, fifty randomly selected X-rays photographs were assessed by a second observer and re-assessed by the first observer after 2 months.

Individual CA was measured by subtracting the calendar birth date of the child from the test date. The CA of participants ranged between 3.1 to 6.6 years, which was classified into six groups for analysis (3.5 years (3.1–3.9), 4.0 years (4.0–4.4), 4.5 years (4.5–4.9), 5.0 years (5.0–5.4), 5.5 years (5.5–5.9), and 6.0 years (6.0–6.6)). Skeletal maturation was expressed as relative SA (SA minus CA), and accelerated skeletal maturation was defined as a relative SA above 1.0 year [9, 12, 13].

### Statistical analysis

Data were entered into an Excel spreadsheet and imported into SPSS Statistics for Mac. Version 21.0 (IBM Co., Armonk, NY, USA) for statistical analysis.

All variables were expressed as mean ± standard deviation (SD). The relative SA was compared with one-way analysis of variance (one-way ANOVA) in BMI groups. The height and weight of the participants were compared with the Standardized Growth Charts for Chinese Children by one-sample t-test. The differences between SA and CA were evaluated by the paired-samples t-test. On the contrary, differences between males and females were analyzed by the independent-samples t-test. Next, the chi-square test was performed to determine statistically significant difference in the frequency of accelerated skeletal maturation in gender, BMI, and age categories. Adjusted odds ratio (OR) and 95% confidence interval (CI) values were obtained from a logistic regression model (for accelerated skeletal maturation) as dependent variables, with gender, age, and BMI levels as predictors. Notably, Goodness-of-fit of the model was estimated using the Hosmer-Lemeshow test.

Reliability analyses of inter- and intra-observer variations were evaluated using the Pearson linear correlation coefficient and Intra-class Correlation Coefficient (ICC), respectively, and notably, the differences were considered statistically significant at *P < 0.05*.

## Results

Concerning inter-observer variations, the Pearson correlation coefficient and ICC were 0.935 (*P < 0.001*) and 0.890 (*P < 0.001*), respectively. On the contrary, the Pearson correlation coefficient and ICC were 0.973 (*P < 0.001*) and 0.894 (*P < 0.001*), respectively, in the intra-observer variations. Notably, all values indicated excellent reliability.

### Characteristics of the participants

The characteristics of participants and the result of one-sample t-test on comparison with Chinese standards are presented in Table 2.

**Table 2:** Participants’ characteristics and the comparison with the Standardized Growth Charts for Chinese Children (urban version, 2015) (Mean ± SD)

		Weight (kg)				Height (cm)			
	N (N1, N2)	Chinese’s Standard	Group 1	Group 2	Total	Chinese’s Standard	Group 1	Group 2	Total
Male									
3.0 ~ < 3.5	40 (0, 40)	15.5 ± 2.0	–	16.3 ± 2.0^*^	16.3 ± 2.0^*^	99.4 ± 4.0	–	99.6 ± 4.1	99.6 ± 4.1
3.5 ~ < 4.0	84 (13, 71)	16.6 ± 2.2	20.2 ± 2.2^**^	17.2 ± 2.5^*^	17.7 ± 2.6^**^	103.2 ± 4.1	104.9 ± 3.9	101.8 ± 4.3^**^	102.3 ± 4.4
4.0 ~ < 4.5	93 (31, 62)	17.8 ± 2.5	20.8 ± 2.5^**^	18.7 ± 2.6^**^	19.4 ± 2.8^**^	106.7 ± 4.2	107.1 ± 3.3	106.9 ± 4.6	106.9 ± 4.2
4.5 ~ < 5.0	151 (41, 110)	19.0 ± 2.8	23.4 ± 3.7^**^	19.1 ± 2.2	20.3 ± 3.3^**^	110.1 ± 4.5	111.8 ± 5.2^*^	109.2 ± 4.1^*^	109.9 ± 4.6
5.0 ~ < 5.5	148 (40, 108)	20.4 ± 3.1	26.2 ± 4.1^**^	20.6 ± 3.1	22.1 ± 4.2^**^	114.1 ± 4.6	116.9 ± 4.4^**^	112.7 ± 4.5^**^	113.9 ± 4.8
5.5 ~ < 6.0	148 (39, 109)	21.7 ± 3.5	27.0 ± 3.2^**^	22.3 ± 4.2	23.5 ± 4.6^**^	117.1 ± 4.7	118.3 ± 4.7	116.2 ± 5.1	116.8 ± 5.1
6.0 ~ < 7.0	66 (37, 29)	23.7 ± 4.0	29.4 ± 4.7^**^	24.4 ± 3.9	27.2 ± 5.0^**^	121.8 ± 4.9	123.5 ± 5.5	119.2 ± 6.2^*^	121.6 ± 6.1
Female									
3.0 ~ < 3.5	41 (0,41)	14.9 ± 1.8	–	15.5 ± 1.9^*^	15.5 ± 1.9^*^	98.3 ± 3.8	–	98.2 ± 3.4	98.2 ± 3.4
3.5 ~ < 4.0	69 (11, 58)	16.0 ± 2.0	19.4 ± 1.7^**^	16.4 ± 1.8	16.9 ± 2.1^**^	102.0 ± 4.0	104.6 ± 5.0	101.1 ± 3.5	101.6 ± 4.0
4.0 ~ < 4.5	83 (21, 62)	16.9 ± 2.2	21.4 ± 3.4^**^	17.1 ± 2.0	18.2 ± 3.1^**^	105.4 ± 4.1	107.9 ± 4.9^*^	104.9 ± 4.7	105.6 ± 4.9
4.5 ~ < 5.0	125 (41, 84)	18.1 ± 2.5	21.8 ± 2.6^**^	18.5 ± 2.4	19.6 ± 2.9^**^	108.9 ± 4.4	109.4 ± 4.3	108.5 ± 4.1	108.8 ± 4.2
5.0 ~ < 5.5	134 (22, 112)	19.5 ± 2.9	25.1 ± 5.2^**^	20.0 ± 2.8^*^	20.9 ± 3.8^**^	112.8 ± 4.5	114.4 ± 6.0	112.3 ± 4.7	112.6 ± 5.0
5.5 ~ < 6.0	106 (20, 86)	20.7 ± 3.2	26.1 ± 3.3^**^	21.8 ± 4.2^*^	22.6 ± 4.4^**^	116.0 ± 4.6	118.6 ± 5.1^*^	115.9 ± 5.1	116.4 ± 5.2
6.0 ~ < 7.0	42 (29, 13)	22.3 ± 3.6	28.9 ± 4.6^**^	23.2 ± 3.5	27.1 ± 5.0^**^	120.2 ± 5.0	122.3 ± 5.2^*^	118.1 ± 5.1	121.0 ± 5.5

Group 1 includes participants with overweight and obesity; Group 2 includes participants with all weights status

*P < 0.05

**P < 0.01 compared with the standard value

In this study, according to the IOTF cut-off points of BMI (for thinness, overweight, and obesity) by gender and age, which is based on international data and linked to the widely acceptable adult cut-off points of BMI (18.5, 25, and 30 kg/m^2^ for male and female) (Table 1), participants were classified into the following groups: thinness group (102, 7.7%), normal-weight group (796, 59.8%), overweight group (excluding children with obesity) (286, 21.5%), and the obese group (146, 11.0%).

The basic information of the study participants is shown in Table 3. The sample consisted of 1330 participants (730 males, 54.9%; 600 females, 45.1%), and no significant difference in CA, SA, and relative SA was observed between males and females; however, the body height, body weight, and BMI were significantly higher in males than females (*P < 0.01*). The results of the paired-samples t-test showed that, overall, the SA was significantly lower than CA in the thinness and normal-weight groups; however, SA was significantly higher than CA in the overweight and obese groups (*P < 0.01*), excluding females with overweight.

**Table 3:** Anthropometric characteristics of the participants in the BMI and gender groups (mean ± SD)

Group	N	CA (years)	SA (years)	Relative SA (years)	BMI (kg/m^2^)	Weight (kg)	Height (cm)
**Total**	1330	4.9 ± 0.8	4.8 ± 1.2^**^	−0.1 ± 0.9	16.8 ± 2.1	20.8 ± 4.6	110.7 ± 7.8
Males	730	4.9 ± 0.8	4.8 ± 1.2^*^	−0.1 ± 0.9	17.0 ± 2.1	21.3 ± 4.7	111.4 ± 7.7
Females	600	4.8 ± 0.8	4.8 ± 1.2^**^	−0.1 ± 0.9	16.5 ± 2.2^##^	20.2 ± 4.5^##^	109.9 ± 7.7^##^
**Thinness**	102	4.8 ± 0.8	4.4 ± 1.2^**^	−0.5 ± 0.9	13.7 ± 0.7	16.6 ± 1.9	110.0 ± 7.0
Males	46	4.8 ± 0.8	4.4 ± 1.3^**^	−0.4 ± 0.9	13.9 ± 0.6	17.0 ± 1.9	110.5 ± 6.9
Females	56	4.8 ± 0.8	4.3 ± 1.2^**^	−0.5 ± 0.9	13.6 ± 0.7^#^	16.3 ± 1.9	109.6 ± 7.0^##^
**Normal**	796	4.7 ± 0.8	4.5 ± 1.0^**^	−0.3 ± 0.8	15.9 ± 0.9	18.9 ± 2.6	108.8 ± 7.1
Males	431	4.8 ± 0.8	4.5 ± 1.1^**^	−0.3 ± 0.8	16.0 ± 0.8	19.2 ± 2.6	109.4 ± 7.0
Females	365	4.7 ± 0.8	4.5 ± 1.0^**^	−0.2 ± 0.8	15.8 ± 0.9^##^	18.5 ± 2.6^##^	108.2 ± 7.1^#^
**Overweight**	286	5.1 ± 0.9	5.3 ± 1.2^**^	0.2 ± 0.9	18.2 ± 0.6	23.5 ± 3.3	113.2 ± 7.6
Males	167	5.1 ± 0.9	5.4 ± 1.2^**^	0.3 ± 0.8	18.4 ± 0.6	23.8 ± 3.5	113.6 ± 7.7
Females	119	5.1 ± 0.8	5.2 ± 1.3	0.2 ± 1.0	18.1 ± 0.5^##^	23.0 ± 3.1^#^	112.6 ± 7.5
**Obese**	146	5.3 ± 0.8	5.8 ± 1.4^**^	0.4 ± 1.1	21.1 ± 1.9	28.7 ± 4.9	116.3 ± 8.0
Males	86	5.3 ± 0.7	5.7 ± 1.3^**^	0.4 ± 1.0	21.1 ± 1.7	29.1 ± 4.5	117.3 ± 7.6
Females	60	5.3 ± 0.8	5.8 ± 1.5^**^	0.5 ± 1.1	21.1 ± 2.0	28.1 ± 5.3	114.9 ± 8.4

*BMI* Body Mass Index, *SD* Standard Deviation, *CA* Chronological Age, *SA* Skeletal Age, relative SA: relative skeletal age (SA - CA). Statistics for SA vs. CA in columns

^*^P < 0.05

^**^P < 0.01

Statistics for males vs. females in rows, ^#^: *P < 0.05*, ^##^: *P < 0.01*

### Skeletal maturation and BMI levels

**Fig. 2:**
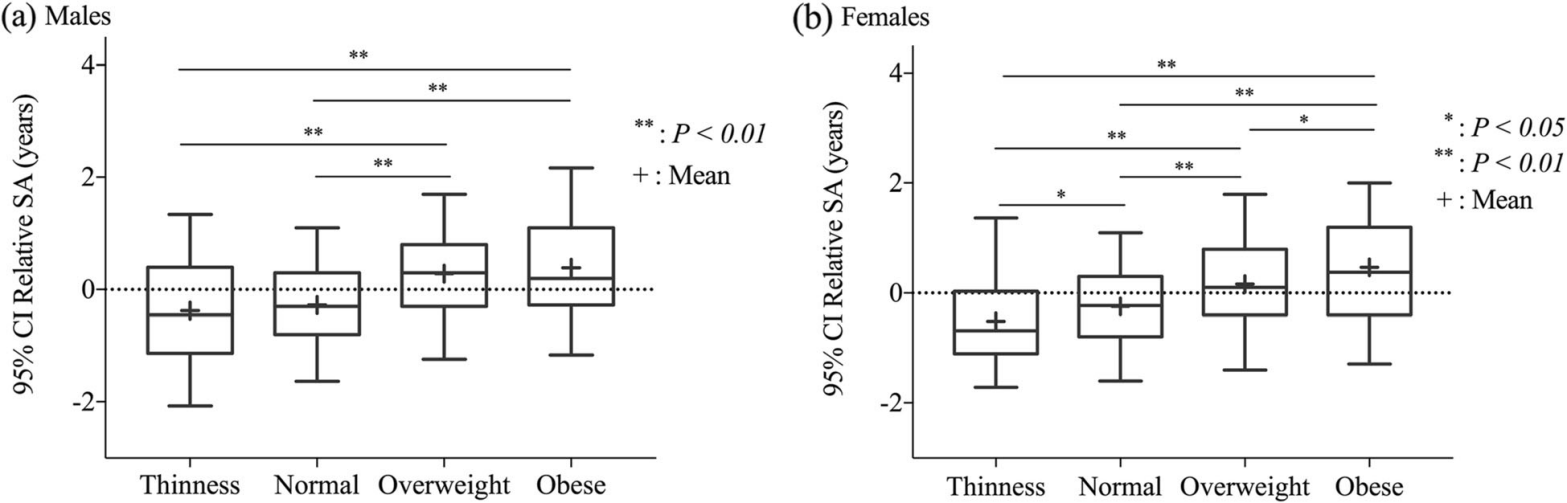
Boxplot of relative skeletal age (SA) according to body mass index (BMI) group in males **(a)** and females (**b**). Greater relative SA indicates earlier maturation. Boxplot shows the median and interquartile ranges; whiskers indicate the range of 95% confidence interval (CI). The difference in significance among groups was examined by one-way analysis of variance (ANOVA) and least significant difference (LSD) post hoc test

As shown in Table 3 and Fig. 2, the trend of relative SA increased with BMI in both gender groups. The one-way ANOVA revealed significant differences in relative SA between BMI groups in both sexes (*P < 0.05*).

**Fig. 3:**
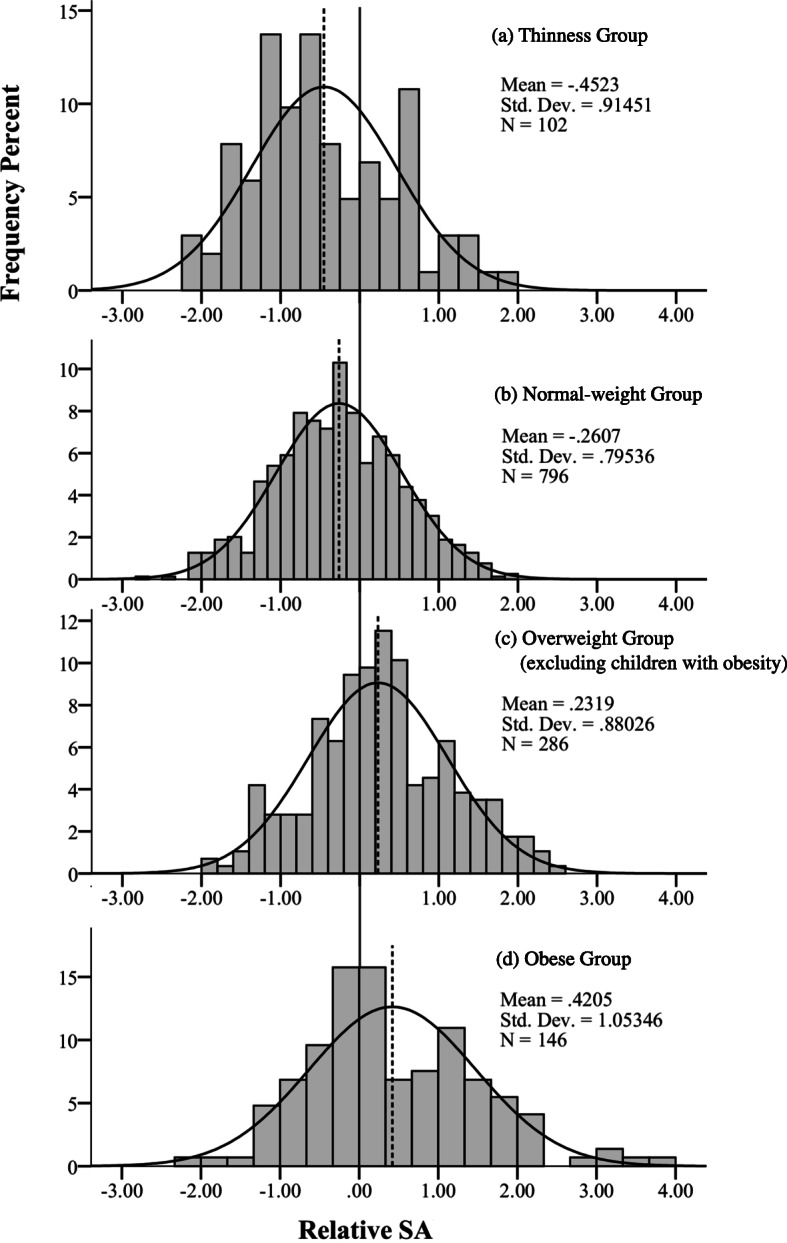
Overlaid histograms of relative SA distributions under different BMI groups. The dotted line represents the mean value of each BMI group: thinness (**a**), normal-weight (**b**), overweight (**c**) and obese (**d**) group. The solid line at position 0 indicates that relative SA = 0 (SA = chronological age [CA]). The solid curve represents the normal distribution

Next, their significance was determined using the least significant difference (LSD) post hoc test. In males, the change in mean relative SA was − 0.5, − 0.3, 0.2, and 0.4 years in the thinness, normal-weight, overweight, and obese groups, respectively. The relative SA distributions in each BMI group are shown in Fig. 3. Although there was no significant difference in the relative SA between the overweight and obese groups, and between the normal weight and thinness groups, the relative SA was significantly greater in both obese and overweight groups than in normal and thinness groups (*P < 0.01*) (Fig. 2a). In females, the post hoc test indicated significant differences in relative SA among all groups in the following order: obese (0.5 years) > overweight (0.2 years) > normal (− 0.2 years) > thinness (− 0.5 years) (*P < 0.01*) (Fig. 2b).

**Fig. 4:**
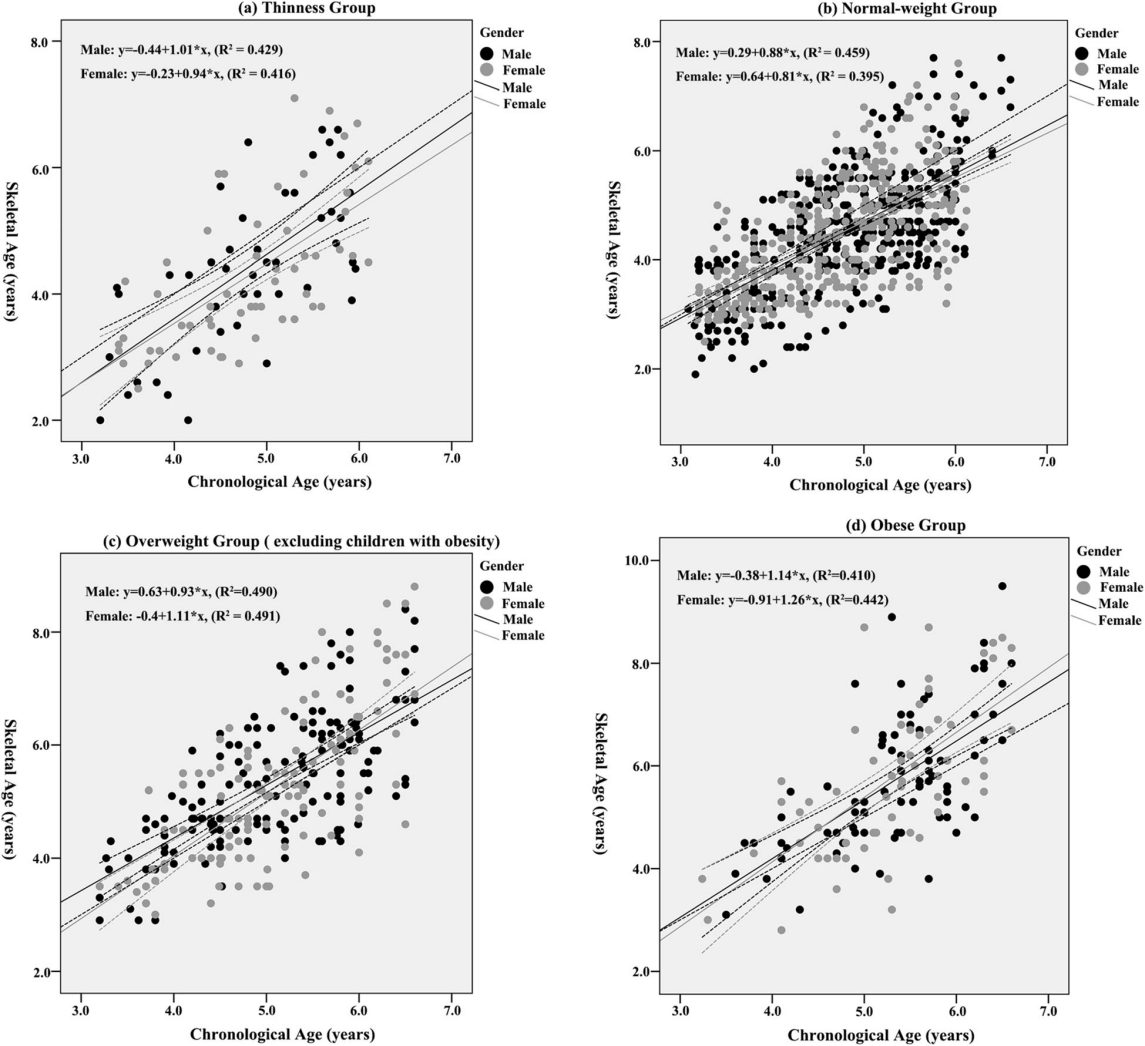
Scatter plots between SA and CA in thinness (**a**), normal-weight (**b**), overweight (**c**), and obese (**d**) groups. Regression lines with 95% CI (the dotted curves) and equations are superimposed on the graph. Black circles and line represent males; grey circles and line represent females; the dotted solid line is the reference line: SA = CA

The relationship between SA and CA is illustrated in Fig. 4. In the thinness and normal-weight groups, SA tended to be lower than CA: 69.6% of children with thinness (29 males, 42 females) (Fig. 4a) and 64.7% of children with normal-weight (284 males, 231 females) (Fig. 4b) had a SA lower than CA. On the contrary, SA tended to be higher than CA in the overweight and obese groups: 58.0% of children with overweight (103 males, 63 females) (Fig. 4c) and 61.0% of children with obesity (52 males, 37 females) (Fig. 4d) had a SA greater than CA. Significant differences in the ratio of SA to CA were observed between the BMI groups (male, *x*^*2*^ *= 48.73, df = 3, P < 0.001*; female, *x*^*2*^ *= 26.22, df = 3, P < 0.01*); however, no differences were observed between males and females. A scatter plot was plotted to visually determine the linear relationship between SA and CA; the regression lines and equations are superimposed on the graph. According to the linear regression model, a greater difference between SA and CA was observed with an increase in CA in the obese group.

### Accelerated skeletal maturation

Table 4 describes the ratio of accelerated skeletal maturation in gender, age, and BMI groups. In this study, 12.9% of males and 13.0% of females had accelerated skeletal maturation. Among the BMI groups, 7.8% of children with thinness, 7.0% of children with normal-weight, 22.0% of children with overweight, and 30.8% of children with obesity had accelerated skeletal maturation (*x*^*2*^ *= 89.442, df = 3, P < 0.01*). In the age groups, the percentage of accelerated skeletal maturation was 3.8% (3.5 years), 13.1% (4.0 years), 10.5% (4.5 years), 13.5% (5.0 years), 16.9% (5.5 years), and 27.8% (6.0 years), respectively (*x*^*2*^ *= 43.417, df = 5, P < 0.01*). No significant differences were observed in the gender groups by the Chi-square test.

**Table 4:** Chi-Square Test for the percentage of relative SA above 1.0 year in the gender, BMI, and age groups

Group	N (%)	x^2^	df	Sig.
Gender				
Male (*n* = 730)	94 (12.9%)	0.000	1	ns
Female (*n* = 600)	78 (13.0%)			
BMI				
Thinness (*n* = 102)	8 (7.8%)	89.442	3	**
Normal (*n* = 796)	56 (7.0%)			
Overweight (*n* = 286)	63 (22.0%)			
Obese (*n* = 146)	45 (30.8%)			
Age ^a^				
3.5 y (*n* = 234)	9 (3.8%)	43.417	5	**
4.0 y (*n* = 176)	23 (13.1%)			
4.5 y (*n* = 276)	29 (10.5%)			
5.0 y (*n* = 282)	38 (13.5%)			
5.5 y (*n* = 254)	43 (16.9%)			
6.0 y (*n* = 108)	30 (27.8%)			

*BMI* body mass index. Relative SA: relative skeletal age (skeletal age -chronological age)

**P < 0.01

^a^3.5 years (3.1–3.9 years), 4.0 years (4.0–4.4 years), 4.5 years (4.5–4.9 years), 5.0 years (5.0–5.4 years), 5.5 years (5.5–5.9 years), 6.0 years (6.0–6.6 years)

In addition, logistic regression analysis was used in the assessment of the effects of age, gender, and BMI groups on the accelerated skeletal maturation of the participants. Consequently, the obtained logistic model had a statistical significance (*x*^*2*^ *= 97.86, P < 0.01)*. According to the Hosmer-Lemeshow goodness-of-fit statistics (*P = 0.812*), the estimated model appropriately fitted 87.1% of the occasions when predicting accelerated skeletal maturation. The results of binary logistic regression analysis of accelerated skeletal maturation are presented in Table 5. After adjusting the two other independent variables (gender and age) in this model, compared with the normal-weight group, participants with overweight/obesity had an increased risk of accelerated skeletal maturation (Overweight, *OR = 3.27, 95% CI: 2.20–4.87*; Obese, *OR = 4.73, 95% CI: 2.99–7.48*).

**Table 5:** Logistic regression analysis of accelerated skeletal maturation (SA – CA ≥1.0 years), adjusting gender and age

Variable	OR (95%CI)	Sig.
BMI		
Normal	1.00 (reference)	
Thinness	1.08 (0.50–2.35)	ns
Overweight	3.27 (2.20–4.87)	**
Obese	4.73 (2.99–7.48)	**

*SA* skeletal age, *CA* chronological age, *CI* confidence interval, *OR* = odds ratio

**P < 0.01

## Discussion

First, the main finding was an association between accelerated skeletal maturation and overweight/obesity existing among preschool children. This result supported the findings of previous research on children and adolescents [15, 17, 18]. Second, 64.7% (515/796) of children with normal-weight had a SA lower than CA (Fig. 4b) in this study, which disagrees with a previous study that encouraged a general advancement with an improvement in the nutritional level of modern people [29]. Third, no statistical difference was observed in relative SA between males and females among children aged 3–6 years (Table 3); this result was inconsistent with a previous conclusion that the development of SA occurs earlier in females than in males from birth to early adolescence [30].

Although previous studies have noted an association between skeletal maturation and obesity in children and adolescents, the results were not uniform. Compared with a previous study, this cross-sectional study was a relatively large sample survey. The results showed that, among Chinese preschool children, the occurrence of accelerated skeletal maturation increased with the BMI: 7.8 and 7.0% of children with thinness and normal-weight developed into 22.0 and 30.8% of children with overweight and obesity, respectively (Table 4). This result strengthened the evidence about the association between accelerated skeletal maturation and overweight/obesity [16, 31, 32]. On the contrary, it indicated that accelerated skeletal maturation might already coexist with overweight/obesity from preschool age, which is worthy of the attention of parents, endocrinologists, and childhood overweight/obesity managers. Skeletal maturation represents a child’s maturation level and height growth space. Children with accelerated skeletal maturation may be observed to have accelerated linear growth; however, the temporary increase in height during childhood would be compensated by an earlier pubertal maturation and a subnormal height gain in adolescence [33–35]. Therefore, accelerated skeletal maturation was considered the leading cause of early fusion of the epiphyseal growth plates, resulting in a compromised adult height [11, 12].

Although there is an association between accelerated skeletal maturation and overweight/obesity, no evidence was found in previous studies regarding an unclear causal relationship between them. Accelerated skeletal maturation and overweight/obesity might interact with each other or correlate with another cardinal factor. It is known that body growth and skeletal maturation during childhood and adolescence are influenced by endocrine and paracrine factors, such as genes, nutrition status, and hormones [14]. Previous studies have shown that overweight/obesity and skeletal maturation are both influenced by these hormones, such as growth hormone (GH) concentration, insulin-like growth factor-1 (IGF-1), estrogens, and androgens [14]. Some evidence suggested that insulin [16] and adrenal androgen [15, 36] play a central role in the association between accelerated skeletal maturation and overweight/obesity in children and adolescents. Based on the evidence, the increased leptin, IGF-1, and sex hormone levels might be implicated in accelerated skeletal maturation in obesity [32]. However, other studies demonstrated no correlation between leptin and skeletal maturation [15, 19]. These previous studies have shown an internal connection between skeletal maturation and obesity, and a combination of multiple hormones affects them.

However, there was no evidence for the mechanism underlying the association between accelerated skeletal maturation and overweight/obesity in preschool children. Based on the previous study that growth and thyroid hormones play the main role in the growth of preschool children and that GH acts mainly through IGF-1 [37, 38], this study speculated from the perspective of hormones that the interaction of GH and IGF-1 might play the most significant role in accelerating skeletal maturation in preschool children with overweight/obesity. The adipose tissue of children with overweight/obesity might lead to an increase in growth hormone and IGF-1, thereby affecting skeletal maturation.

Another consideration was that nutritional status was an important factor influencing both accelerated skeletal maturation and overweight/obesity. Several studies indicated that the secular trend of earlier maturation could be caused by improved nutrition and socioeconomic environments [29, 38–40], which is the same reason for the increasing prevalence of childhood overweight and obesity worldwide [2, 41]. Conversely, as shown in the results, 69.6 and 64.7% of children with thin and normal-weight had lower SA than CA, respectively (Fig. 4a, b). The result, which is consistent with the evidence that 83.33% of malnourished subjects showed delayed SA [42], could be explained by insufficient nutrient intake. Concerning the result that children with normal weight had lower SA than CA, a longitudinal study that significant differences in BMI emerged earlier (by age 2 to 5 years) than relative SA could be an explanation [43]. This might indicate that nutrition has a faster impact on BMI than skeletal maturation and that both greater BMI and nutritional affection accelerate skeletal maturation among preschool children.

The results of this study showed that among preschool children aged 3–6 years, height, weight, and BMI were significantly higher in males than females (which was consistent with previous studies) [44]. Regarding skeletal maturation, previous studies indicated an increasing gender difference [45] from birth (female’s skeletal maturation being 4–6 weeks more advanced than males) [30] to early adolescence (at a difference of 1.9 years) [46]. However, our results revealed no gender differences in skeletal maturation among children aged 3 to 6 years. Therefore, it was speculated according to the result mentioned earlier, that skeletal maturation is related to BMI. In this study, the overall BMI was significantly higher in males than in females; this might balance the early development of females described in a previous study. As a result, no difference was observed in the average relative SA between males and females.

This study has some limitations related to the standard of TW3-C RUS, based on TW3, which was employed in the assessment of skeletal maturation [27]. A previous study showed that there are different SA values between three commonly used methods of SA assessment −TW methods, Greulich-Pyle, and Fels methods [47]. The results of this study might not fully be applicable to studies using other methods of SA assessment. Additionally, the reference sample of TW3-C RUS was based on Chinese children and adolescents. These may affect the generalizability of the results to children of different races in the population and even children from other provinces in China. In addition, despite BMI, which was used as the indicator of overweight/obesity, strongly correlating with the direct measures of body fat (body fat percentage, skinfold-thickness) [48–50], the study findings still cannot convincingly explain the direct association between accelerated skeletal maturation and adipose tissue. Another minor limitation is that our study was conducted among Han Chinese children. The racial differences in growth and development should be considered when citing the results of this study to other study groups or populations.

## Conclusions

This study provided evidence that there is an association between accelerated skeletal maturation and overweight/obesity among preschool children. This suggests that accelerated skeletal maturation might coexist with overweight/obesity in preschool children, and interventions, such as dietary modifications and increasing levels of physical activity [51, 52], should be employed to prevent both accelerated skeletal maturation and overweight/obesity as early as preschool age.

## Data Availability

The datasets used and analyzed during the current study are available from the corresponding author on reasonable request.

## Abbreviations

BMI: Body Mass Index
SA: Skeletal Age
CA: Chronological Age
IOTF: International Obesity Task Force
OR: Odds Ratio
CI: Confidence Interval
WHO: World Health Organization
TW3-C RUS: Tanner–Whitehouse 3-Chinese Radius–Ulna–Short bones
SD: Standard Deviation
ANOVA: Analysis of Variance
ICC: Intra-class Correlation Coefficient
LSD: Least Significant Difference
GH: Growth Hormone
IGF-1: Insulin-like Growth Factor-1
